# Construction and applications of SARS-CoV-2 pseudoviruses: a mini review

**DOI:** 10.7150/ijbs.59184

**Published:** 2021-04-10

**Authors:** Minghai Chen, Xian-En Zhang

**Affiliations:** 1 CAS Key Laboratory of Quantitative Engineering Biology, Shenzhen Institute of Synthetic Biology, Shenzhen Institutes of Advanced Technology, Chinese Academy of Sciences, Shenzhen 518055, China; 2 National Laboratory of Biomacromolecules, CAS Center for Excellence in Biomacromolecules, Institute of Biophysics, Chinese Academy of Sciences, Beijing 100101, China

**Keywords:** COVID-19, SARS-CoV-2, pseudovirus, spike protein, packaging system

## Abstract

The ongoing coronavirus disease 2019 pandemic, caused by severe acute respiratory syndrome coronavirus 2 (SARS-CoV-2), has posed a serious threat to global public health and social stability. There is an urgent need for understanding the nature and infection mechanism of the virus. Owing to its high infectivity and pathogenicity and lack of effective treatments, live SARS-CoV-2 has to be handled in biosafety level 3 laboratories, which has impeded research into SARS-CoV-2 and the development of vaccines and therapeutics. Pseudotyped viruses that lack certain gene sequences of the virulent virus are safer and can be investigated in biosafety level 2 laboratories, providing a useful virological tool for the study of SARS-CoV-2. In this review, we will discuss the construction of SARS-CoV-2 pseudoviruses based on different packaging systems, current applications, limitations, and further explorations.

## Introduction

The outbreak of coronavirus disease 2019 (COVID-19) has caused major global human health and social upheaval. According to the World Health Organization (WHO), as of February 23, 2021, more than 111 million cases and 2.4 million deaths have been confirmed worldwide, with numbers continuing to increase on a daily basis (https://covid19.who.int/). Severe acute respiratory syndrome coronavirus 2 (SARS-CoV-2), a new member of the genus *Betacoronaviruses* and a close relative of SARS-CoV, has been identified as the cause of the global pandemic^1^-^3^. Studies indicate that both SARS-CoV-2 and SARS-CoV utilize angiotensin-converting enzyme 2 (ACE2) as the host cell surface receptor to gain entry into target cells^4^. SARS-CoV-2 is a positive-strand RNA virus with a 30 kb genome^5^. It has a round or elliptic morphology, with a diameter of approximately 60-140 nm, and is characterized by a crown-like appearance under electron microscopy observation owing to the presence of spike glycoproteins on the envelope. The first two-thirds of the genome, named the ORF1ab region, encodes 16 non-structural proteins. The rest of the genome encodes several accessory proteins and four structural proteins, including spike (S) glycoprotein, envelope (E), matrix (M), and nucleocapsid (N) proteins, which are important for maintaining the structural integrity of the enveloped SARS-CoV-2 virion^6^-^8^.

Studies of live SARS-CoV-2 are restricted to biosafety level 3 laboratories, which has made SARS-CoV-2 research inaccessible to the majority of research laboratories around the world, which would seriously delay the development of vaccines and therapeutics against SARS-CoV-2 if there were no alternative solution. Pseudoviruses are a kind of recombinant virus with their core or backbone and surface proteins derived from different viruses^9^. Genes inside pseudoviruses are usually altered or modified to abolish native surface protein expression. An additional plasmid is then used to express alternative surface proteins, producing a pseudovirus that can infect susceptible host cells but can only replicate intracellularly for a single round^10^, ^11^. As viral surface proteins play pivotal roles in gaining entry into host cells, the conformational structures of pseudoviral surface proteins have high similarity to that of the native viral proteins; however, pseudoviruses have attenuated virulence compared with wild-type (WT) viruses, allowing them to be safely handled in biosafety level 2 laboratories^12^. As a result, pseudoviruses are widely used for the study of cellular tropism^13^, receptor recognition^14^, drug discovery^15^, and for developing and evaluating antibodies and vaccines^16^, ^17^. In this review, we discuss the current development and applications of SARS-CoV-2 pseudotyped viruses, their limitations, and further development directions.

## Packaging systems for SARS-CoV-2 pseudoviruses

### The human immunodeficiency virus (HIV-1)-based lentiviral packaging system

**Figure 1:**
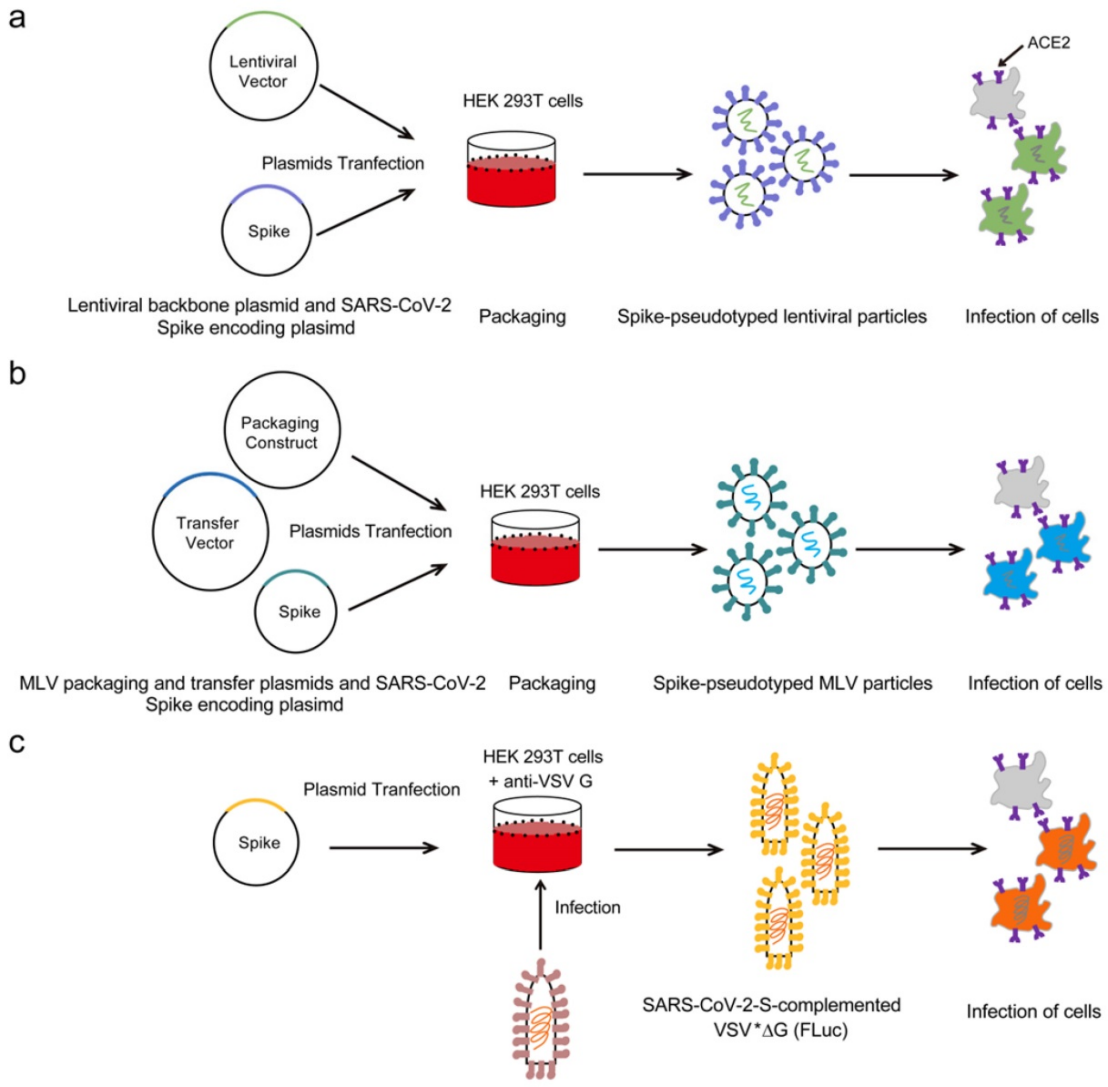
**The strategies to acquire SARS-CoV-2 pseudoviruses based on different packaging systems**. (a) HEK 293T cells were transfected with a plasmid encoding lentiviral backbone and a plasmid expressing SARS-CoV-2 Spike. The transfected cells produced Spike-pseudotyped lentiviral particles and these viral particles can infect cells that express the ACE2 receptor. (b) HEK 293T cells were co-transfected with an Spike encoding-plasmid, an MLV Gag-Pol packaging construct and the MLV transfer vector encoding a luciferase reporter. The transfected cells produced Spike-pseudotyped MLV particles and these viral particles can infect cells that express the ACE2 receptor. (c) HEK 293T cells were transfected with SARS-CoV-2 Spike expression plasmid, after 24 h post-transfection, the cells were inoculated with VSV*∆G (Fluc) encoding firefly luciferase. After an incubation period of 1h at 37 ℃, the inoculum was removed and cells were washed with PBS before medium supplemented with anti VSV-G antibody was added in order to neutralize residual input virus. Spike-pseudotyped particles were harvested 20 h postinoculation and could infect cells that express the ACE2 receptor.

The HIV-1 lentiviral packaging system is currently the most widely used SARS-CoV-2 pseudoviral packaging system. **Figure 1a** shows the basic strategies to generate the SARS-CoV-2 pseudoviruses based on the HIV-1-derived lentiviral particle. In general, two or three plasmids are co-transfected into the cells to produce the pseudoviruses. The two-plasmid packaging system is the most widely used SARS-CoV-2 pseudoviral packaging system, which includes a plasmid to express the SARS-CoV-2 S protein and another HIV-1 backbone plasmid to express the packaging proteins and signals but with the envelope gene deleted. Several HIV-1 lentivial backbone plasmids are used in the two-plasmid packaging system, such as pNL4-3-kfs^18^ and pNL4-3.Luc.R^-^E^-^19-21. The HIV-1 three-plasmid packaging system is usually comprised of a packaging plasmid, a transfer plasmid containing the reporter gene, and a SARS-CoV-2 S protein-expressing plasmid. Specifically, this system involves splitting the HIV‐1 backbone into separate packing and transfer plasmids. The packaging plasmid expresses the Gag and Pol proteins, while the transfer plasmid contains the *cis*‐regulatory elements needed for HIV-1 reverse transcription, integration, and packaging, as well as multiple cloning sites and a reporter gene under the control of a heterogeneous promoter^22^, ^23^. The SARS-CoV-2 S protein-expressing plasmid is made of a vector carrying the S gene driven by a cytomegalovirus (CMV) promoter. Several independent groups have reported that co-transfection of pNL4-3.Luc.R^-^E^-^ and a SARS-CoV-2 S protein-expressing plasmid can produce SARS-CoV-2 pseudoviruses^15^, ^19^-^21^. Our group produced pseudotyped viruses through co-transfection of pNL4-3-kfs and a SARS-CoV-2 S protein-expressing plasmid (unpublished). Ou et al co-transfected a SARS-CoV-2 S protein-expressing plasmid, psPAX2, and pLenti-GFP into HEK 293T cells to produce SARS-CoV-2 pseudoviruses^23^. Yan et al co-transfected a pCDH plasmid harboring SARS-CoV-2 specific genes, psPAX, and pMD2G into the HEK 293T cells to successfully produce SARS-CoV-2 pseudoviruses^24^.

### The murine leukemia virus (MLV)-based packaging system

MLVs are typical simple retroviruses, they are enveloped and contain positive-strand RNA bearing three genes (*gag*, *pol* and *env*) that encode viral capsid proteins, viral enzymes and envelope proteins, respectively^25^. Early work by Witte and colleagues showed that when vesicular stomatitis virus (VSV) is used to infect cells in which MLV is packaged, pseudoviruses can be harvested for use in neutralization antibody assays, indicating its potential as a packaging system for the production of pseudotyped viruses^26^. **Figure 1b** shows the strategies to generate the SARS-CoV-2 pseudoviruses based on the MLV packaging system. Recently, Zheng et al co-transfected MLV packaging system containing three plasmids into HEK 293FT cells to generate SARS-CoV-2 pseudovirion particles: the first plasmid was the packaging construct, SV-Psi^-^-Env^-^-MLV; the second plasmid was L-LUC-SN, a minimal retroviral transfer vector encoding the luciferase reporter gene; and the third plasmid was an expression construct encoding the SARS-CoV-2 S protein^27^. Walls et al co-transfected an MLV Gag-Pol packaging construct, a MLV transfer vector encoding a luciferase reporter, and an S protein-encoding plasmid into the HEK 293T cells to successfully produce SARS-CoV-2 pseudoviruses^28^.

### The VSV packaging system

VSV is a negative-strand RNA virus that possesses a single type envelope glycoprotein (G) mediating its entry^29^. Previous studies have shown that G-deleted VSV can be complemented in *trans* with the envelope glycoproteins of even unrelated viruses, including coronaviruses^30^, ^31^. Stillman et al cloned the VSV genome into a plasmid to make stable VSV, which was subsequently used to generate pseudoviruses carrying heterogeneous glycoproteins^32^. Various reporter genes were successively inserted into this plasmid to facilitate the detection of infected cells in a reasonable period of time^33^. **Figure 1c** shows the outlines to generate the SARS-CoV-2 pseudoviruses based on the VSV packaging system. Briefly, HEK 293T cells were grown in cell culture dishes and transfected with SARS-CoV-2 spike protein expression plasmid, after 24 h post-transfection, the cells were washed and inoculated with VSV*∆G (Fluc) encoding firefly luciferase. After an incubation period of 1h at 37 ℃, the inoculum was removed and cells were washed with PBS before medium supplemented with anti VSV-G antibody was added in order to neutralize residual input virus. Pseudotyped particles were harvested 20 h postinoculation. Recently, Zettl et al co-transfected a pseudotyped VSV*∆G (FLuc), a G-deleted VSV encoding both GFP and firefly luciferase, with the SARS-CoV-2 S protein and used it for the detection of SARS-CoV-2 neutralizing antibodies in the sera of convalescent COVID-19 patients^17^. Letko et al used VSV∆G-luc bearing SARS-spike chimeras to study cell entry and receptor usage for SARS-CoV-2 and other lineage B betacoronaviruses^34^. Nie et al successfully constructed a pseudovirus neutralization assay for SARS-CoV-2, consisting of a pseudotyped VSV bearing the full-length spike protein of SARS-CoV-2^35^. Notably, when the VSV packaging system is used to make the SARS-CoV-2 pseudovirus, there may be residual VSV virus mixed with the pseudovirus, which may interfere with the neutralization assay by producing false-positive results. Preferably, the amount of VSV should be minimized; however, if excess VSV is believed to be interfering with a pseudovirus-based assay, treatment of the pseudovirus preparation with a VSV neutralizing antibody could be considered^36^.

### Other packaging systems

Besides the aforementioned SARS-CoV-2 pseudovirus packaging systems, researchers have also produced SARS-CoV-2 pseudoviruses with other packaging systems. Yan et al used a phage vector to produce a SARS-CoV-2 pseudovirus. Specifically, an MS2 vector was used to insert SARS-CoV-2 genes for N, E, and ORF1ab^24^. In general, while packaging 500 bp of RNA is efficient using MS2, packaging 1.5 kb of RNA does not work well^37^. Although MS2-based armored L-RNA technology can package >2 kb RNA sequences^38^, the packaging capacity of MS2 is still not as good as lentiviruses. Owing to this limitation, genes for N, E, and nine parts of ORF1ab gene were inserted into the MS2 vector separately, and different MS2-based pseudoviruses were mixed together to form quality control materials. Researchers also used a NS1-deleted influenza A virus as the vector to express SARS-CoV-2 Spike protein, and this influenza virus-based pseudovirus is now being tested as candidate vaccine in a clinical trial^39^. The adenovirus vector was used to express SARS-CoV-2 S protein and being evaluated as candidate vaccine against SARS-CoV-2^40^-^42^.

## Applications of SARS-CoV-2 pseudoviruses

### Mechanistic study of viral infection and entry

Pseudoviruses have been widely used for conducting *in vitro* studies on the interaction between viruses and host cells^43^. They have also proven to be very useful for *in vivo* studies, particularly on the mechanism of viral infection and biodistribution^44^. Reporter genes are usually incorporated into pseudoviruses to easily perform quantitative analyses. These mainly include luciferase and fluorescent protein-encoding genes, with each option displaying particular advantages and disadvantages. For example, bioluminescence assays usually have lower background noise and are more sensitive, but data acquisition and analysis can time-consuming and expensive. In contrast, assays using a fluorescent protein as the indicator are much cheaper and easier to operate; however, they may face higher background noise issues and are less sensitive^45^-^47^. Along with the aforementioned study in Letko et al, Hoffmann and colleagues used pseudotyped SARS-CoV-2 particles to show that SARS-CoV-2 infection depends on the host cell factors ACE2 and transmembrane protease, serine 2 (TMPRSS2)^36^. Li et al recovered rVSV-eGFP-SARS-CoV-2 particles and confirmed that the pseudovirus displayed entry characteristics similar to that of the WT virus, such as cell tropism and pH-dependence^48^. Ou et al used a lentiviral pseudotype system to determine cell type susceptibility, virus receptor, and entry pathway for SARS-CoV-2^23^. Yi et al utilized a spike-containing SARS-CoV-2 pseudovirus to infect neural layers within human embryonic stem cell-derived brain organoids and monolayer cortical neurons, revealing that mature neurons express ACE2 at the soma and are susceptible to invasion of the spike-pseudotyped SARS-CoV-2, establishing an in vitro model to study the impact of SARS-CoV-2 on the human brain^49^. Some examples of the applications of SARS-CoV-2 pseudoviruses are listed in **Table 1**.

### Quantification of neutralizing antibodies

Currently, the spike-containing SARS-CoV-2 pseudovirus is the mostly developed pseudoviral system. As various studies have verified that SARS-CoV-2 uses ACE2 as the host cell surface receptor, and the interaction between the S protein and ACE2 mainly mediates its entry to the target cells^4^, ^28^, thus, antibody neutralization assays target S protein based on SARS-CoV-2 pseudoviruses have been widely investigated. Compared with traditional assays, the reported pseudovirus-based assays have demonstrated good correlation with WT virus-based assays, while also typically being higher throughput and requiring less sample serum^57^. Zettl et al reported a VSV-based SARS-CoV-2 pseudovirus and used it to determine neutralizing antibody titers in convalescent COVID-19 patients, showing that most patients had only low titers of neutralizing antibodies (50% neutralizing dose < 320)^17^. Nie et al evaluated neutralizing antibodies against SARS-CoV-2 in biosafety level 2 facilities by using a VSV pseudovirus production system^35^. Crawford et al used pseudotyped lentiviral particles to measure the neutralizing activity of human sera or plasma against SARS-CoV-2 in a luciferase-based assay, providing a valuable complement to enzyme-linked immunosorbent assay-based methods that measure antibody binding rather than neutralization^50^.

### Vaccines approach

Pseudovirus-based candidate vaccines against SARS-CoV-2 are also under development. These candidate vaccines with features of live attenuated vaccine which induces robust immune responses including mucosal immunity. The adenoviral vectors based pseudoviruses are the most commonly used vaccines against SARS-CoV-2, such as adenovirus type 5^40^, adenovirus type 26^41^, and ChAdOx^42^. All of these candidate vaccines are now currently being tested in phase III clinical trials. The researchers of University of Hong Kong used a influenza A virus-based pseudovirus to develop SARS-CoV-2 vaccine, which is now in a clinical trial^39^. Case et al reported a live attenuated, replication-competent, VSV vector based vaccine protected against SARS-CoV-2-mediated pathogenesis in mice^56^. Researchers of Shenzhen Geno-Immune Medical Institute engineered the dendritic cells (DC) with the lentiviral vector expressing the SARS-CoV-2 structural proteins and protease to develop the candidate vaccine, which named LV-SMENP-DC^39^. In addition, measles virus expressing SARS-CoV-2 S was generated and used as a live attenuated vaccine, while not all immunized animals develop neutralizing activity detectable in the assay^58^.

### Inhibitor screening

Screening of small‐molecule inhibitors against SARS-CoV-2 has also been performed using SARS-CoV-2 pseudoviruses. Yang et al used pseudotyped SARS-CoV-2 to screen an approved drug library of 1,800 small molecular drugs for SARS-CoV-2 virus entry inhibitors. Fifteen active drugs were identified as specific SARS-CoV-2 S pseudovirus entry inhibitors, and further antiviral tests using native SARS-CoV-2 virus in Vero E6 cells confirmed that seven of these drugs significantly inhibited SARS-CoV-2 replication, reducing supernatant viral RNA load with a promising level of activity^15^. Puhl et al repurposed Ebola and Marburg virus inhibitors tilorone, quinacrine, and pyronaridine for SARS-CoV-2 based on the SARS-CoV-2 pseudovirus^59^. Zhu et al used a lentiviral-based pseudotyped SARS-CoV-2 particle to evaluate potent membrane fusion inhibitors^20^. Besides the aforementioned SARS-CoV-2 entry small-molecule inhibitors screenings, efforts have been made into evaluating SARS-CoV-2 main protease and MEK inhibitors^51^, ^53^.

### Other applications

Guo et al used a pseudovirus with the SARS-CoV-2 S protein as a model and confirmed that plasma-activated water (PAW) effectively inhibited pseudovirus infection through S protein inactivation^60^. Yan et al compared seven commercial SARS-CoV-2 nucleic acid detection reagents with pseudovirus as the quality control material and found that a pCDH-based pseudovirus was more similar to SARS-CoV-2 than an MS2-based pseudovirus, and was therefore more suitable for evaluating SARS-CoV-2 nucleic acid test kits^24^. Our group also made a lentiviral vector-based SARS-CoV-2 pseudovirus, combining it with a fluorescence labeling technique for real-time imaging of single SARS-CoV-2 entry in respiratory epithelial cells (unpublished).

## Limitations and weakness

There are several issues that should be considered with SARS-CoV-2 pseudoviral systems. SARS-CoV-2 pseudoviruses typically only contain the S protein of WT SARS-CoV-2, which can largely mediate viral entry in a fashion similar to that of the WT virus. Because SARS-CoV-2 pseudoviruses can only replicate for a single round and may not always induce pathogenesis as their WT counterparts do, results from assays using pseudotyped viruses should be compared and validated against WT virus-based assays that remains the gold standard^61^. Additionally, the virus shape may influence its suitability for constructing a corresponding pseudotyping virus. For example, HIV-1 and MLV are spherical viruses, whereas VSV is bullet-shaped. Therefore, the pattern of S protein distribution/conformation/density on a pseudotyped virus may not reflect the “natural” state of S proteins on SARS-CoV-2. It is necessary to use various packaging systems to simultaneously prepare pseudoviruses^62^, ^63^. Currently, the S incorporated pseudotyped viruses were the mainly developed SARS-CoV-2 pseudoviruses, which were unable to conduct researches on functions and processes related to other viral proteins. In addition, to develop pseudoviruses-based candidate vaccines, researchers should consider the pre-existing immunity to the viral vectors. A phase I trial of an Ad5-nCoV demonstrated the result of diminishing vaccine efficiency in individuals with high pre-existing Ad5 immunity^40^. Moreover, the pseudoviruses-based candidate vaccines worked as live attenuated viruses may face the issues about genome instability which may lead to a back mutation recovering their virulence.

## Further explorations

The currently developed SARS-CoV-2 pseudoviruses only contain the SARS-CoV-2 S protein, which restrict their applications; a pseudovirus with multiple viral components of SARS-CoV-2 will partly solve the issues, our group is now trying to alter or modify the viral genome to make a SARS-CoV-2 pseudovirus which contains multiple viral components. Genetic code expansion technology has been used to generate a live but replication-incompetent influenza A virus^64^, and such strategies could be considered for the construction of SARS-CoV-2 pseudoviruses.

## Figures and Tables

**Table 1:** SARS-CoV-2 pseudoviruses

Virus Protein	Packaging System	Application	Reference
SARS-CoV-2 Spike	pCMVdeltaR8.91, pLAS2w.RFP-C.Pneo, pMD.G-S	Neutralization antibody assay	22
SARS-CoV-2 Spike	HDM-IDTSpike-fixK, HDM-Hgpm2, HDM-tat1b, pRC-CMV-Rev1b, pHAGE-CMV-Luc2-IRES-ZsGreen-W	Neutralization antibody assay, Main protease inhibitors evaluation	50, 51
SARS-CoV-2 Spike	psPAX2, pLenti-GFP, pCMV14-3×Flag-S	Virus entry and immune cross-reactivity, identifying monoclonal antibodies	23, 52
SARS-CoV-2 Spike (D614/G614)	pHAGE-fullEF1α-ZsGreen-IRES-Puro(R), Lentiviral proteins encoding plasmids (Tat, Rev and Gag/Pol), pS (D614/G614)	MEK inhibitors evaluation	53
SARS-CoV-2 Spike	pcDNA3.1-SARS2-Spike, psPAX2, pUltra-hot	Brain organoids infection	49
SARS-CoV-2 Spike	pNL4-3.Luc.R^-^E^-^, pcDNA3.1-SARS-CoV-2-S or pCMV3-SARS-CoV-2-S	Neutralization antibody assay and entry inhibition test, entry inhibitors identification, membrane fusion inhibitors evaluation	15, 19-21
SARS-CoV-2 Spike	pNL4-3/KFS, pSARS-CoV-2-S	Virus assembly	18
SARS-CoV-2 Spike	pCMV-MLVgag-pol, pTG-Luc, pCAGGS-SARS-CoV-2-S	Virus entry and cross-neutralizing antibody assay	28, 54
SARS-CoV-2 Spike	SV-Psi--Env--MLV, L-LUC-SN, pSARS-CoV-2-S	Neutralization assay	27
SARS-CoV-2 Spike	rVSV∆G-G, pSARS-CoV-2-S	Entry and neutralization assay, vaccine	17, 35, 36, 48, 55, 56
SARS-CoV-2 ORF1ab, N, E	psPAX, PCDH-ORF1ab-N-E, pMD2G; MS2-ORF1ab, MS2-N, MS2-E	Comparing commercial SARS-CoV-2 nucleic acid detection reagents	24
SARS-CoV-2 Spike	NS1-deleted influenza A virus, pSARS-CoV-2-S	vaccine	39
SARS-CoV-2 Spike	adenovirus type 5, adenovirus type 26, ChAdOx, SARS-CoV-2-S	vaccine	40-42
